# Hypocomplementemia during tocilizumab treatment: Long-term follow-up results

**DOI:** 10.1097/MD.0000000000029528

**Published:** 2022-06-17

**Authors:** Amir Bieber, Doron Markovits, Kohava Toledano, Yonit Tavor, Reuven Mader, Alexandra Balbir-Gurman, Yolanda Braun-Moscovici

**Affiliations:** a Rheumatology Unit, Ha’Emek Medical Center, Afula, Israel; b B Shine Rheumatology Institute, Rambam Health Care Campus, Haifa, Israel; c Rappaport Faculty of Medicine, Technion, Haifa, Israel.

**Keywords:** hypocomplementemia, rheumatoid arthritis, interleukins, tocilizumab

## Abstract

Hypocomplementemia has been reported in patients with rheumatoid arthritis treated with tocilizumab (TCZ), but its long-term consequences are unknown. We assessed the long-term outcome of patients treated with TCZ who developed hypocomplementemia regarding serious bacterial infections or autoimmune diseases (AID).

The charts of patients treated with TCZ at two rheumatology centers were reviewed retrospectively. Data regarding patients’ age, gender, disease duration, autoantibodies status, previous or concomitant treatments, blood counts, liver enzymes, C3 and C4 levels at baseline and during TCZ treatment, episodes of infections, allergic reactions, and AID were analyzed. Univariate analysis was used to compare patients with low C3, C4 levels versus patients with normal C3, C4 levels. Variables that were statistically significant associated or tended to be associated with low C3 or C4 were included in multiple variable logistic regression.

Of 132 patients treated with TCZ, 108 had serial measurements of serum complement concentration. Thirty-three (30%) patients developed low C4 levels and 23 (21%) had also low C3. Mean TCZ treatment period was 4.9 years (range, 1–14 years). All patients had normal complement levels at baseline. Leukopenia occurred in 18 (16.7%) patients, 14 of whom (77%) had low complement. Persistent leukopenia was observed in 8% and 5.3% of patients with normal C3 and C4 levels, respectively, as opposed to 47% and 42% of patients with low C3 or low C4, respectively. Low C3, C4 levels correlated with prolonged TCZ treatment retention time and effectiveness. There were no serious bacterial infections or new onset AID.

Hypocomplementemia during TCZ treatment was accompanied by leukopenia that correlated with treatment duration. Hypocomplementemia was not associated with serious bacterial infections or new onset AID. Decreased complement levels were associated with treatment longevity. The role of monitoring complement level in predicting treatment response or assessing disease activity deserves further investigation.

## 1 Introduction

Interleukin-6 (IL-6) is a key cytokine involved in the pathogenesis of rheumatoid arthritis as a component of a broad spectrum of the inflammatory cytokine family which acts upon pathologically participating cells.^[1,2]^ On target cells, IL-6 interacts with its receptor complex, consisting of the IL-6 receptor (IL-6R) and two molecules of gp130 expressed on the cell membrane.^[3]^ Phosphorylation of the intracellular portion of gp130 results in subsequent control of Janus Kinase (JAK) intracellular signal transducer and activator of transcription (STAT) STAT1 and STAT3 activity, which leads to upregulation of inflammatory genes.^[4]^ In an active inflammatory state, IL-6 and IL-1 mediate the production of C-reactive peptide (CRP) and other acute phase proteins and the increase in erythrocyte sedimentation rate (ESR).^[5–7]^

Complement system activation plays a crucial role in the pathogenesis of systemic lupus erythematosus (SLE), small vessel vasculitis, cryoglobulinemia, sepsis, bacterial endocarditis, and others.^[8]^ Some of these illnesses are associated with complement consumption and reduction of serum complement levels. On the other hand, congenital low levels of C3 and C4 were associated with development of SLE, membranoproliferative glomerulonephritis, pyogenic, and Neisseria infections.^[9]^

Blockage of IL-6 receptor with TCZ leads to rapid reduction in inflammatory markers, and accordingly in a substantial portion of Rheumatoid Arthritis (RA) and vasculitis patients leads to clinical and laboratory improvement and disease control.^[10,11]^

Complement levels were so far found to be normal or high in patients with RA,^[12]^ Juvenile Idiopathic Arthritis (JIA),^[13]^ Takayasu Arteritis (TAK),^[14]^ and Giant Cell Arteritis (GCA),^[15]^ and usually correlated with disease activity. In each of these, levels were at the normal range of C3 90 to 180 mg/dL and of C4 10 to 40 mg/dL or higher. Decline of complement levels were reported in lupus patients and in patients with RA treated with TCZ.^[10,16–18]^ The precise mechanism of complement reduction under TCZ therapy is unknown. Decreased complement (C3, C4) production was suggested as a possible mechanism.^[10]^

Interestingly, treatment with other biological drugs was not associated with complement levels reduction.

The long-term effects of complement levels reduction by TCZ have not yet been investigated. Our aims were to assess the long-term outcome of patients treated with TCZ who developed hypocomplementemia regarding bacterial infections, newly developed autoimmune diseases (AID) or other safety issues, and the possible impact on drug retention.

## 2 Methods

### 2.1 Patients

The charts of 132 consecutive patients treated with TCZ at two rheumatology centers (Rheumatology Institute at Rambam Health Care Campus, Haifa, and Rheumatology Unit at Ha-Emek Medical Center, Afula) from January 2005 until December 2018 were reviewed retrospectively.

The inclusion criteria were: TCZ treatment for at least six months, baseline complement blood levels (C3, C4) and two or more serial measurements of complement blood levels under TCZ treatment.

The exclusion criteria were: Patients lost for follow-up, patients without results of C3, C4 levels and/or without serial tests for leucocyte, thrombocyte count and liver enzymes.

The indications for TCZ treatment were: RA, juvenile idiopathic arthritis, adult onset Still's disease, Takayasu's arteritis, GCA, Systemic Sclerosis (SSc), and mixed connective tissue disease (MCTD). All patients met the classification criteria and diagnosis for each disease: RA was diagnosed according to ACR-EULAR 2010 classification criteria,^[19]^ JIA according to International League Against Rheumatism (ILAR) criteria,^[20]^ adult onset Still's disease according to Yamaguchi's criteria,^[21]^ Takayasu arteritis according to ACR 1990 criteria for Takayasu vasculitis^[22]^ and GCA according to ACR 1990 criteria for giant cell arteritis,^[23]^ systemic sclerosis according to 2013 classification criteria for systemic sclerosis^[24]^ and MCTD according to Alarcón-Segovia criteria.^[25]^ The baseline dose of TCZ was intravenous 8 mg/kg every 4 weeks (maximal dose 800 mg) (124 patients) or subcutaneous 162 mg/week (8 patients).

The study was approved by the Ethics Committee of Rambam Health Care Campus (0252-10RMB) and of Ha-Emek Medical Center (018-17EMC).

The study was retrospective and informed consent was not required.

### 2.2 Data extraction

The following data were extracted from patients’ files: age, gender, diagnosis, disease duration, Clinical Disease Activity Index (CDAI) (for patients with RA and JIA), presence of rheumatoid factor (RF), anti-citrullinated protein antibodies (ACPA) and antinuclear antibodies (ANA), previous and concomitant non-biological Disease Modifying Anti Rheumatic Drugs (DMARDs), previous biological DMARDs, TCZ treatment duration, dose, and the reason for TCZ discontinuation. TCZ retention or survival time measured the length of time until discontinuation of the drug. Tested laboratory parameters included hemoglobin, leucocyte and thrombocyte count, liver enzymes (performed monthly for the first 6 months and afterwards every 2–3 months), and C3 and C4 levels at baseline and during TCZ treatment (every 6 months). Leukopenia was defined as white blood cell count <4.2 × 109/L (according to local lab). Complement levels were measured using immunoturbidimetry method. The normal range of C3 was 90 to 180 mg/dL and of C4 10 to 40 mg/dL. Low complement levels were defined as C3 <90 mg/dL and/or C4 <10 mg/dL. Only patients who had results of C3, C4 levels at baseline (before starting TCZ) and at least 2 more measurements during TCZ treatment, were included in the study.

Reported episodes of significant infections, hypersensitivity reactions, and de novo AID were captured and analyzed. Significant infections were considered as such if they required antibiotic therapy, hospitalization, temporary discontinuation of TCZ or opportunistic. Hypersensitivity reaction was defined as occurrence of rash/urticaria, shortness of breath, angioedema, or anaphylactic reactions during or within 24 hours of infusion or injection of TCZ. De novo AID was defined as new-onset lupus or membranoproliferative glomerulonephritis.

### 2.3 Statistical analysis

All statistical tests were 2 sided, statistical significance was defined as *P* value below .05. Categorical variables were summarized as frequency and percentage. Continuous variables were evaluated for normal distribution using histogram and Q–Q plots and reported as median and interquartile range. Association between continuous variables was evaluated using Spearman correlation. Association between categorical variables was evaluated using Chi square test or Fisher exact test. The association with continuous variables was studied with independent sample *t* test or Mann–Whitney *U* test.

Univariate analysis was used to compare patients with low C3, C4 levels versus patients with normal C3, C4 levels, while controlling for potential confounders. Variables that were statistically significant associated or tended to be associated with low C3 or C4 were included in multiple variable logistic regression. Odds ratio and 95% confidence interval were reported. SPSS version 25 was used for the statistical analysis.

## 3 Results

One hundred and eight patients fulfilled the inclusion criteria of the study. [RA (112 patients), JIA (8), adult Still's disease (4), Takayasu's arteritis (4), GCA (2), SSc (1) and MCTD (1)].

Twenty-four patients were excluded: eight patients discontinued TCZ within less than six months of treatment or were lost to follow up; five patients had no C3 and C4 results at all, and 11 patients had one measure only of complement blood levels.

The patients on intravenous tocilizumab underwent monthly physician and patient activity disease assessment when receiving the treatment at the rheumatology day-care centers of the 2 hospitals. The patients on subcutaneous tocilizumab were followed and assessed every 3 months at the rheumatology clinics in both centers. Patients’ demographic, clinical and laboratory data are presented in Table 1. Median follow-up was 4.9 years (range, 1–14 years). Forty-six (43%) patients were naïve to biologicals. Thirty-five (32%) patients were on concomitant prednisone treatment (median dose 5 mg/daily). All the patients included in the study had results of complement levels prior to tocilizumab initiation and every 6 to 12 months afterwards. At baseline, all patients had normal C3 and C4 levels, while 11 patients had positive ANA. During follow-up, 38 (35%) patients developed low complement levels (C3 < 90 mg/dL, C4 < 10 mg/dL): Thirty-three (30%) had low levels of C4, 23 (21%) had low C3 and low C4 levels. Female patients developed low levels of C3 more frequently than did men (*P* = .043).

**Table 1:** Demographic and clinical parameters of tocilizumab-treated patients.

	C3 normal – 84 pts	C3 low 24 pts	*P*	C4 normal – 75 pts	C4 low – 33 pts	*P*
Age (years)	53.6 ± 13.7	54.2 ± 12.8	=.85	53.2 ± 13.3	54.9 ± 12.8	=.55
Gender						
FemaleMale	54 (64.3%)30 (35.7%)	20 (87%)3 (13%)	=.043	51 (68%)24 (32%)	23 (69.7%)10 (30.3%)	=1.00
Patients on previous Biologics-number (%) (yes)	47 (57.3%)	12 (52.2%)	=.81	41 (56.2%)	19 (57.6%)	=1.00
MTX treatment	46 (54.8%)	13 (56.5%)	=1.00	45 (60.0%)	15 (45.5%)	=.21
Prednisone	30 (36%)	5 (20%)	=.1	18 (24%)	12 (36%)	=.06
CDAI mean (SD)	8.5 (5.7)	6.7 (3.3)	=.2	8.7 (5.6)	6.2 (2.9)	=.07
Treatment retention time^∗^ (years)	4.13 ± 3.37	5.88 ± 3.32	=.020	4.28 ± 2.89	5.73 ± 3.99	=.049
No of patient with Leukopenia n, (%)	7 (8.3%)	11 (47.8%)	<.0001	4 (5.3%)	14 (42.4%)	<.001
ANA baseline (negative)	78 (92.9%)	18 (78.3%)	=.056	69 (92.0%)	28 (84.8%)	=.31
ANA during TCZ treatment (negative)	73 (89.0%)	19 (82.6%)	=.48	66 (90.4%)	27 (81.8%)	=.22

ANA = antinuclear antibody, CDAI = Clinical Disease Activity Index, MTX = methotrexate, TCZ = tocilizumab.

∗ Treatment retention time—the length of time until discontinuation of the drug (tocilizumab).

No new AID occurred in patients with low complement levels and there was no predisposition to severe bacterial infections (median follow-up −4.9 years). TCZ retention time correlated with occurrence of low C3 and C4 (*P* = .020, *P* = .049) (Table 1). Patients with low C3 tended to discontinue treatment less often due to inefficacy or allergic reactions. Statistically significant correlation was found between the occurrence of leukopenia and the development of low complement C3 and C4 levels (*P* < .0001 and *P* < .001, Table 1). Leukopenia (white blood cell count < 4.2 × 109/L) occurred in 18 (16.7%) patients; C3 and C4 were low in 11 and 14 of these patients, respectively. Almost half the patients with low complement levels developed leukopenia (median [IQR] 3.6 K/μL [3.0–4.1]). The differential count of leukocytes was normal, except for 2 patients who had mild neutropenia (1.4 K/μL). There was no correlation between complement levels and prednisone, methotrexate, previous DMARDs and biologicals. Changes in ANA status did not correlate with complement levels. Borderline association between baseline positive ANA to low C3 development was observed, but not to C4 (small number of patients). None of the ANA positive patients developed lupus.

Multivariate analysis provided significant correlations between low C3, C4 levels and leukopenia (*P* < .001 and .0001, respectively), and between low C3, C4 and treatment retention time (*P* = .020, *P* = .049) (Tables 2 and 3).

**Table 2:** Correlation between complement level and Age, Gender, Treatment retention time, and Leukopenia.

					95% CI for odds ratio	
		*B*	*P*	Odds ratio	Lower	Upper
Step 1	Age	0.007	.748	1.007	0.967	1.048
	Gender	0.755	.286	2.127	0.532	8.513
	Treatment retention time^∗^	2.869	.0041	3.778	1.524	9.367
	Leukopenia	2.051	.001	7.773	2.361	25.587
	Constant	−3.417	.010	0.033		

Multivariant analysis for C3. Variables in the equation.

∗ Treatment retention time—the length of time until discontinuation of the drug (tocilizumab).

**Table 3:** Correlation between complement level and Age, Gender, Treatment retention time and Leukopenia.

					95% CI for odds ratio	
		*B*	*P*	Odds ratio	Lower	Upper
Step 1	Age	0.016	.396	1.016	0.979	1.054
	Gender	−0.706	.183	0.494	0.175	1.396
	Treatment retention time^∗^	3.346	.0008	5.217	1.982	13.731
	Leukopenia	2.789	.000	16.258	4.296	61.524
	Constant	−2.255	.044	0.105		

Multivariant analysis for C4.

∗ Treatment retention time—the length of time until discontinuation of the drug (tocilizumab).

**Figure 1:**
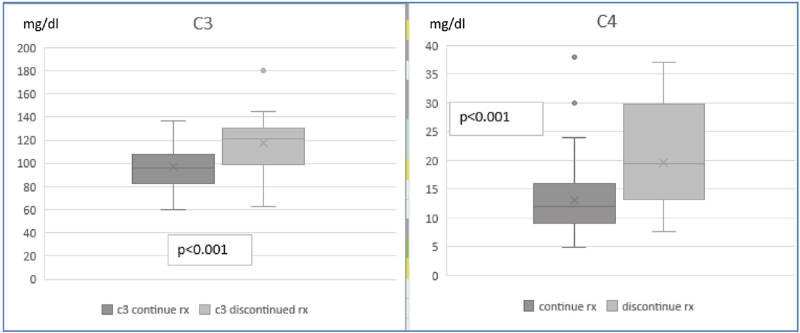
Correlation between C3, C4 serum level and drug retention. C3 levels: normal range 90–180 mg/dL. C4 levels: normal range 10–40 mg/dL. Rx = tocilizumab treatment.

Highly significant correlations were found between serum levels of C3 and C4 to drug retention time (*P* < .001, Fig. 1). Complement levels were normal in 90% of patients who discontinued treatment for secondary failure. The odds ratio for patients with low C3 or C4 to continue TCZ were 3.778 (95% CI 1.524–9.367) and 5.217 (95% CI 1.982–13.731) (Tables 2 and 3). Forty-two of the 108 patients discontinued TCZ. Reasons for discontinuation are listed in Table 4. The patient with malignancy of breast, was a 63-year female patient, who received TCZ for 5 years. No other cases of malignancy occurred. The decisions to change the therapeutic regimen were taken based upon clinical assessment of disease activity, irrespective of complement levels. Complement levels normalized in all patients who discontinued TCZ within 1 to 3 months.

**Table 4:** Reasons for TCZ discontinuation.

Reason for discontinuation	Number of patients	Specification
Secondary inefficacy	16	Median treatment duration 3.5 years, range 1–5 years
Side effects/intolerance	11	Allergic reactions
	2	Recurrent elevated liver function tests
	2	Pneumonitis and Pneumonia
	1	Aortic aneurysm
	1	Corneal ulcer
	1	Crohn's exacerbation
	1	Abdominal pain
	1	Carcinoma of breast
Other reasons (non-medical)	13	Non-medical
Total	42	

## 4 Discussion

A significant subset of TCZ treated patients in our rather large cohort developed low complement levels—35%. TCZ-induced hypocomplementemia was observed not only among RA patients but also in patients with different AID. Our primary aim was to assess the long-term outcome of patients treated with TCZ who developed hypocomplementemia, regarding bacterial infections, AID or other safety issues. During a relatively long follow-up, none of the patients from our cohort, with low complement levels, developed new AID, nor had severe bacterial infections. The secondary aim of our study was to evaluate whether low complement levels may have an impact on the TCZ retention time. Indeed, we found a significant correlation between low C3, C4 levels and prolonged treatment retention time (univariate analysis, Table 1 and multivariate analysis Tables 2 and 3, Fig. 1). Ninety percent of patients who discontinued TCZ due to secondary inefficacy had normal complement levels and leukocyte count. The decisions to discontinue TCZ were taken based upon clinical assessment of disease activity, which was taken by the treating rheumatologist, irrespective of complement levels. Nevertheless, we did not find a difference regarding disease activity measured as CDAI (Clinical Disease Activity Index) for RA patients, of which this study was not powered to. Hypocomplementemia was more frequent in female patients (C3) and was associated with the occurrence of leukopenia (Table 2). The association between leukopenia and low complement was confirmed by multivariate logistic regression analysis (Tables 2 and 3, Fig. 1). We did not find any correlation between complement levels and treatment with prednisone, methotrexate, previous DMARDs and biologicals. We did not find an increased prevalence of malignancy in our cohort, considering that our study was not powered for that purpose.

A reduction of complement levels following TCZ treatment (although within normal limits) was reported in the OPTION study^[10]^ and later by other authors, including us, in a previous study on a smaller cohort.^[16–18,26]^ Romano et al described a reduction of C3, C4 levels below normal range in 11/16 and 9/16 RA patients during TCZ treatment. The complement levels remained low during further drug administration. Reduction in complement levels under TCZ treatment correlated with improvement of RA activity measured by RA disease activity score (DAS28).^[18]^ The median follow-up was 38 months, shorter than our study.

The synthesis of serum complement components occurs mainly in the liver by hepatocytes and, less frequently, by other cells, such as fibroblasts, monocytes, and astrocytes.^[27–29]^ The complement system works synergistically with Toll-Like receptors (TLR), mostly TLR type 4, to upregulate pro-inflammatory cytokines of the IL-6 family.^[30]^ It is reasonable to assume that the systemic inflammatory process up-regulates IL-6 production by immune cells via TLR, complement receptors and other innate immune alarm receptors. TCZ inhibits IL-6 signaling through a competitive blockade of the IL-6 binding site and can bind to both sIL-6R and mIL-R6, thus inhibiting both classic signaling and trans signaling in cells that express mIL-6R or gp130, respectively.^[31]^ TCZ treatment results in very fast lowering of acute phase reactants, mainly CRP, through blocking of downregulation production signals in affected hepatocytes; similarly, complement factors production is downregulated through those mechanisms by IL-6 blockade. It seems that hypocomplementemia represents decreased production rather than increased complement system activation and consumption.^[16]^

As the complement system is a central part of the immune system, there might be concerns regarding association between low complement levels and an increased rate of infections, new-onset AID, or malignancy among TCZ treated patients. Our study did not reveal increased incidence of infections, AID, or malignancy among patients with low complement levels. IIlei et al found no correlation between complement levels and increased rate of infections, in a study on lupus TCZ treated patients.^[16]^

New-onset glomerulonephritis with positive dsDNA and low complement levels was reported in a RA patient treated with TCZ.^[32]^ Other studies did not find an increased incidence of new onset AID in TCZ treated patients.^[16,18,26]^ Likewise, no increased rates of malignancy were found in TCZ-treated RA patients,^[33]^ supporting our data.

TCZ treatment is associated with occasional neutropenia, but the underlying mechanisms of this aberration are unclear. A study examining the influence of TCZ on neutrophils function in vitro did not find increased apoptosis or phagocytosis of neutrophils.^[34]^ Another study did find an impaired leukocyte trafficking to bone marrow, or function impairment.^[35]^ A study by Moots et al, found that RA treated with TCZ had a higher rate of neutropenia, not resulting in increased infections rates.^[36]^ In our study we did find an association between leukopenia and low complement levels, which might represent an up-stream effect of IL-6 blockade. In this real life cohort, we noticed only mild leukopenia with normal differential count except for 2 patients with mild neutropenia.

The strengths of our study are the prolonged follow-up (mean of 59 months, range of 12–168 months), the relatively high number of patients with high-quality monthly assessment and the inclusion of patients with diverse autoimmune/autoinflammatory diseases and not only RA.

Limitations of this study: Being a retrospective study and the exclusion of 24 patients from the analyses due to lack of complete data are the main limitations of the study. Also, TCZ retention was calculated based on the treating rheumatologist retrospectively, and not according to a prospective study protocol. Another limitation is that we included in our study patients with different indications for TCZ therapy, yet we assumed that the low complement levels were not associated with the indication itself. Complement levels were measured within the first 6 months of TCZ treatment and afterwards every 6 months. Most of the patients with low complement levels, showed a reduction of complement within the first 18 months of treatment, so we do believe that the hypocomplementemia was not related to the duration of treatment. Altogether we cannot rule out completely a reverse casuality.

In conclusion, a decrease of complement levels is quite frequent among TCZ-treated patients. The scarcity of new onset AID and the low incidence of severe infections during the rather long-term follow-up of our cohort is reassuring. The association of low complement levels with treatment retention and a lower rate of treatment discontinuation might reflect the highly potent anti-inflammatory effect of TCZ.

In view of the inherent difficulty to assess the inflammatory activity in patients treated with TCZ because its inhibition on IL6 signaling and consequently the lowering of acute phase reactants, the role of monitoring complement level in predicting treatment response or assessing disease activity deserves further investigation.

The relationship between leukopenia and low complement levels, in the context of TCZ treatment needs further elucidation.

## Author contributions

**Conceptualization:** Doron Markovits, Reuven Mader, Yolanda Braun-Moscovici.

**Data curation:** Kohava Toledano, Yolanda Braun-Moscovici, Yonit Tavor, Amir Bieber.

**Formal analysis:** Yolanda Braun-Moscovici.

**Investigation:** Yolanda Braun-Moscovici, Yonit Tavor, Amir Bieber.

**Methodology:** Doron Markovits.

**Project administration:** Yolanda Braun-Moscovici.

**Resources:** Kohava Toledano, Yonit Tavor, Amir Bieber.

**Supervision:** Alexandra Balbir-Gurman, Reuven Mader, Doron Markovits.

**Validation:** Yonit Tavor.

**Writing – original draft:** Amir Bieber, Yolanda Braun-Moscovici.

**Writing – review & editing:** Alexandra Balbir-Gurman, Doron Markovits, Reuven Mader, Yolanda Braun-Moscovici.
